# Perioperative neurocognitive disorders: a comprehensive review of terminology, clinical implications, and future research directions

**DOI:** 10.3389/fneur.2025.1526021

**Published:** 2025-08-26

**Authors:** Xuli Ren, Lian Huiqiao, Yuntu Wu, Te Zhang, Peng Chen, Longyun Li, Guoqing Zhao, Fang Wang

**Affiliations:** ^1^Jilin University, Changchun, China; ^2^Department of Anesthesiology, The Third Hospital of Jilin University, Changchun, Jilin, China; ^3^School of Basic Medicine, Jilin Medical University, Changchun, Jilin, China; ^4^Department of Anesthesiology, Jilin People's Hospital, Jilin, China; ^5^Changchun Jirun Jingyue Hospital, Changchun, Jingyue, China; ^6^Rehabilitation Department of Mongolian Hospital in Kuleun Banner, Tongliao City, Inner Mongolia Autonomous Region, China

**Keywords:** perioperative neurocognitive disorders, postoperative cognitive dysfunction, neurocognitive recovery, perioperative risk factors, delirium diagnosis, surgical outcomes, cognitive rehabilitation

## Abstract

Perioperative neurocognitive disorders (PNDs) encompass a spectrum of cognitive impairments that may affect patients before, during, or after surgical procedures, with significant implications for patient outcomes, and healthcare expenditures. This comprehensive review examines the evolution of PND terminology, clinical manifestations, diagnostic criteria, incidence rates, risk factors, underlying mechanisms, preventive measures, management strategies, and future research directions. The authors emphasize the importance of early diagnosis and intervention to enhance postoperative recovery and underscore the necessity of a multidisciplinary approach to patient care.

## 1 Introduction

Perioperative neurocognitive disorders (PNDs) encompass a spectrum of cognitive impairments that may affect patients before, during, or after the surgical procedure. The conceptualization and nomenclature of PNDs have undergone significant evolution, with an increasing emphasis on early diagnosis, prevention, and intervention, to improve patient outcomes. PNDs comprise a range of Cognitive impairment can manifest at various stages of the surgical process (1). These include pre-existing cognitive impairment, delirium occurring up to seven days post-surgery, delayed neurocognitive recovery (diagnosed up to 30 days post-surgery), and postoperative neurocognitive disorder diagnosed thereafter, until 12 months. The Diagnostic and Statistical Manual of Mental Disorders, 5th edition (DSM-5) provides the criteria for diagnosing these disorders (2).

PNDs can significantly impair the quality of life of patients and their ability to perform daily activities. Moreover, they may increase morbidity, mortality, and health care expenditures. Early identification of PNDs facilitates timely intervention, potentially mitigating cognitive decline and improved postoperative recovery (3). Strategies for prevention and treatment encompass avoiding potential contributors, implementing non-pharmacological and pharmacological interventions, and utilizing anesthetics with a reduced cognitive impact. The evolution of PNDs terminology and understanding has resulted in a more comprehensive approach to diagnosing and managing these disorders, ultimately enhancing patient care quality.

## 2 Historical evolution of the PNDs terminology

Various factors have influenced the development of PNDs, including advancements in medical studies, enhanced understanding of cerebral anatomy and physiology, and the establishment of diagnostic criteria, and assessment tools.

**Figure 1:**
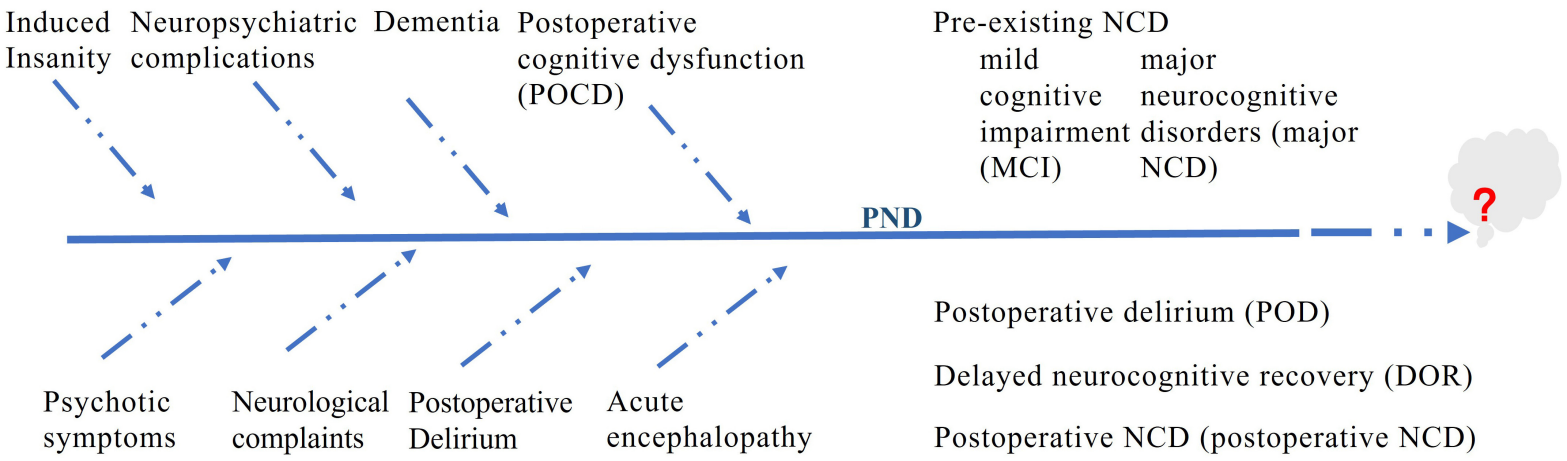
Evolution of terminology for perioperative neurocognitive disorders.

We conducted a comprehensive literature review to identify studies on PNDs. The search methodology was meticulously crafted to reflect the historical progression, underlying mechanisms, and clinical ramifications described in this manuscript. The primary databases used were PubMed, Web of Science, and Google Scholar. The search terminology encompassed PND-related phrases such as “perioperative neurocognitive disorders,” “postoperative cognitive dysfunction,” “postoperative delirium,” “delayed neurocognitive recovery,” “anesthetic neurotoxicity,” “cognitive impairment,” “postoperative cognitive,” as well as “POCD” and “POD.” Only articles published in English were considered. Table 1 highlights the key studies on PNDs. Figure 1 shows the changes in the terminology of PNDs.

**Table 1:** Summary of key studies related to PNDs.

**References**	**Study**	**Methodology**	**Key findings**	**Contributions to the field**
Savage (4)	Insanity Following the Use of Anæsthetics in Operations	Case reports documenting psychiatric symptoms following anesthesia.	Chloroform and other anesthetics may induce psychiatric complications post-surgery.	Early recognition of anesthesia-related cognitive and psychiatric disturbances
Turville and Dripps (71)	The anesthetic management of the aged	Observational study on anesthesia management in elderly patients.	Elderly patients are more susceptible to adverse effects of anesthesia, including cognitive decline.	Highlighted the need for tailored anesthesia management in older adults.
Bedford (5)	Adverse cerebral effects of anesthesia on old people	An observational study documenting cognitive and psychiatric complications in elderly patients after anesthesia.	Anesthesia, particularly in older adults, can lead to cognitive decline and psychiatric symptoms.	Highlight the potential cognitive risks of anesthesia in elderly patients, laying the groundwork for future research on POCD.
Papper (72)	Anesthesia in the aged	Review of anesthesia effects in elderly patients.	Elderly patients are more vulnerable to cognitive decline post-anesthesia due to reduced physiological reserve.	Emphasized the importance of considering age-related physiological changes in anesthesia management.
Simpson et al. (73)	The effects of anesthesia and elective surgery on old people	Observational study on cognitive outcomes in elderly patients after surgery.	Elderly patients experience significant cognitive decline after surgery, particularly with longer procedures.	Highlighted the long-term cognitive risks associated with surgery in older adults.
Kornfeld et al. (74)	Psychiatric complications of open-heart surgery	Observational study on psychiatric outcomes after cardiac surgery.	Open-heart surgery is associated with a high incidence of psychiatric complications, including delirium and cognitive decline.	First to link cardiac surgery with psychiatric and cognitive complications, influencing future research on POCD.
Shaw et al. (75)	Neurologic and neuropsychological morbidity following major surgery	Comparative study of cognitive outcomes after coronary artery bypass (CABG) and peripheral vascular surgery.	CABG is associated with higher rates of neurologic and neuropsychological morbidity compared to peripheral vascular surgery.	Highlighted the differential cognitive impact of surgical procedures, particularly cardiac surgery.
Murkin et al. (76)	Statement of consensus on assessment of neurobehavioral outcomes after cardiac surgery	Consensus statement on neurobehavioral outcomes after cardiac surgery.	Standardized assessment tools are needed to evaluate cognitive outcomes after cardiac surgery.	Provided a framework for standardized cognitive assessment in PND research.
Moller et al. (77)	Long-term postoperative cognitive dysfunction in the elderly ISPOCD1 study	Longitudinal study on long-term cognitive outcomes in elderly patients after surgery.	25.8% of elderly patients experienced long-term cognitive dysfunction after non-cardiac surgery.	Established the concept of long-term POCD and identified age as a significant risk factor.
Van Dijk et al. (78)	Cognitive outcome after off-pump and on-pump coronary artery bypass graft surgery	Randomized trial comparing cognitive outcomes after off-pump vs. on-pump CABG.	Off-pump CABG was associated with better short-term cognitive outcomes compared to on-pump CABG.	Highlighted the role of surgical technique in cognitive outcomes, influencing the adoption of off-pump techniques.
Selnes et al. (79)	Cognitive outcomes 3 years after coronary artery bypass surgery	Longitudinal study comparing cognitive outcomes after CABG vs. non-surgical controls.	CABG patients showed no significant difference in cognitive outcomes compared to non-surgical controls at 3 years.	Challenged the notion that CABG causes long-term cognitive decline, emphasizing the need for further research.
Monk et al. (80)	No improvement in neurocognitive outcomes after off-pump vs. on-pump coronary revascularisation	Meta-analysis comparing neurocognitive outcomes after off-pump vs. on-pump CABG.	No significant difference in neurocognitive outcomes between off-pump and on-pump CABG.	Suggested that surgical technique alone may not be the primary determinant of cognitive outcomes.
Monk et al. (81)	Predictors of cognitive dysfunction after major noncardiac surgery	A prospective study identifying predictors of cognitive dysfunction after non-cardiac surgery.	Age, education level, and pre-existing cognitive impairment are significant predictors of postoperative cognitive dysfunction.	Identified key risk factors for PNDs, informing preoperative risk assessment and intervention strategies.
Evered et al. (9)	Recommendations for the Nomenclature of Cognitive Change Associated with Anesthesia and Surgery	Review and consensus-based recommendations for standardizing PND terminology.	The proposed updated nomenclature includes preoperative NCD, postoperative delirium (POD), delayed neurocognitive recovery (DNR), and postoperative NCD.	Provided a standardized framework for diagnosing and classifying PNDs, facilitating better communication and research consistency.
Ren et al. (22)	Dysfunction of the Glymphatic System as a Potential Mechanism of Perioperative Neurocognitive Disorders	Review of the role of the glymphatic system in PNDs, focusing on waste clearance, neuroinflammation, and anesthesia effects.	Glymphatic dysfunction, caused by anesthesia, surgery, and sleep disturbances, contributes to PNDs by impairing waste clearance and promoting neuroinflammation.	Introduced the glymphatic system as a key mechanism in PNDs, emphasizing its role in waste clearance and neuroinflammation and suggesting potential therapeutic targets.
Feng et al. (28)	Association between cerebrovascular disease and perioperative neurocognitive disorders	A retrospective cohort study investigated the relationship between pre-existing cerebrovascular disease (CVD) and PNDs.	CVD was an independent risk factor for PNDs, with an odds ratio of 10.193.	Demonstrated a strong association between cerebrovascular disease and PNDs, emphasizing the importance of preoperative vascular health assessment.

Early medical literature documented cognitive and mental disorders associated with surgical procedures, or anesthesia. In 1887, Dr. Savage, a British physician, recorded cases of severe psychosis following operations and hypothesized that anesthetic substances, particularly chloroform, and can potentially induce insanity (4).

Before the 1980s, cognitive changes following surgery were often attributed to normal aging processes or considered a side effect of anesthesia, without specific terminology. Confusion or delirium after surgery is prevalent; however, these conditions lack precise definitions (5). Systematic documentation of these conditions emerged in the late 19th and early 20th centuries, respectively. Researchers have identified a subset of patients, predominantly Older adults exhibit cognitive decline postoperatively. This observation has prompted increased attention and research on the enduring cognitive effects of surgical intervention. Terminology such as “anesthesia-induced delirium” or “postoperative confusion” was adopted to characterize the mental alterations associated with surgical procedures and anesthesia administration (6).

As cognitive impairments beyond those immediately following anesthesia have been recognized, and the terminology has evolved to encompass this broader spectrum. The mid-20th century saw the introduction of modern anesthesia, enabling surgeons to conduct more intricate operations, such as heart bypass surgeries. This advancement subsequently heightened recognition of Cognitive issues after surgery (7). The terms “postoperative cognitive dysfunction” (POCD) and “postoperative delirium” (POD) were coined to describe the decline in cognitive abilities of surgical patients. POCD was initially used to describe cognitive deterioration following surgery, particularly in elderly patients. Acute changes in attention and cognitive function characterize the development of postoperative delirium. These two terms are commonly employed in the scientific literature to refer to cognitive impairments that manifest after surgery and persist for different durations.

As the 20th century progressed, the field of postoperative cognitive change expanded in terms of its terminology. A new classification system, “postoperative neurocognitive disorders”, was established, encompassing both delirium and cognitive dysfunction. This development introduced a more refined nomenclature to accurately describe the cognitive alterations observed in patients following surgical procedures (8). During this period, research has focused on identifying the risk factors, elucidating the underlying mechanisms, and developing preventive strategies.

In recent years, the definition of PNDs has been expanded to include a broader spectrum of cognitive deficits that may occur not only after surgery but also before or during the procedure (9). The evolution of terminology reflects an enhanced understanding of how cognitive alterations can manifest throughout the perioperative process. This updated nomenclature more accurately portrays the range of cognitive disorders and acknowledges that these changes may occur throughout the surgical trajectory, from preoperative evaluation to postoperative recuperation. The scope of this term has been expanded to include PNDs, encompassing cognitive changes that can manifest before, during, and after surgery, including delirium. PNDs now recognize delirium as a crucial element with potentially enduring implications.

Significant publications and classification systems, particularly the Diagnostic and Statistical Manual of Mental Disorders (DSM) and the World Health Organization's International Classification of Diseases (ICD), have played crucial roles in shaping the terminology and comprehension of PNDs throughout history. These key resources have markedly influenced the development of these concepts over time (2).

The DSM has defined diagnostic criteria for neurocognitive disorders since 1952, with editions refining these classifications. Notably, the DSM-III introduced a standardized framework, and the DSM-5, released in 2013, has significantly influenced the understanding of PNDs. The DSM-5 categorizes these disorders into major and mild neurocognitive disorders and delirium, with specific criteria for each category. The DSM-5 classifies delirium according to its causes, aiding the identification of PNDs for targeted prevention and treatment (9).

In addition, the International Classification of Diseases (ICD) plays a crucial role in shaping the terminology and understanding of PNDs. For instance, ICD-10 contains categories for cognitive disorders relevant to PNDs, including dementia and memory disorders. The most recent version, the ICD-11, incorporates codes for cognitive disorders associated with surgical procedures, thereby improving the global classification and acknowledgment of these conditions (10). The ongoing development of ICD has influenced the international classification and acknowledgment of cognitive disorders related to surgical procedures.

The nomenclature for PNDs has progressed from early descriptive terms to a more comprehensive label, reflecting an expanded and sophisticated understanding of the various cognitive impairments linked to surgical procedures. This evolution in terminology has been influenced by advancements in medicine, modifications in diagnostic standards, and the growing recognition of the significance of cognitive functions in patient care. As research progresses, we anticipate that our understanding and terminology will continue to develop and capture the intricacies of these disorders more accurately in the future. Terminology will evolve as our knowledge deepens and new diagnostic tools and criteria emerge.

## 3 Clinical manifestations and diagnosis

PNDs exhibit diverse clinical features involving a wide array of cognitive, behavioral, and emotional alterations that can substantially affect a patient's recovery after surgery. The cognitive aspects of PNDs include impaired memory, confusion, reduced mental processing speed, and challenges in focusing and maintaining attentiveness. Behavioral symptoms may include restlessness, increased irritability, lack of interest, and hostility. Emotional manifestations can range from feelings of anxiety and depression to unpredictable mood changes and emotional instability. These symptoms can manifest immediately after surgery to several months post-surgery and may persist with varying intensity (11).

The detection of PNDs is complex and requires clinical evaluation, patient history, and cognitive assessments without a definitive standard. The DSM-5 defines PNDs as cognitive decline with significant functional impairments that cannot be explained by other disorders and occur post-surgery (9, 12). The following four categories of PNDs are defined according to the DSM-5 (Table 2):

**Preoperative NCD:** Cognitive impairment before surgery. Based on their severity, these impairments can be further classified as mild or major, affecting the patient's capacity to perform activities of daily living.
**Postoperative delirium (POD) is** characterized by acute and fluctuating disturbances in attention, awareness, and cognition that manifest rapidly. It is typically identified within the first week post-surgery and necessitates thorough evaluation to differentiate it from other cognitive alterations.
**Delayed neurocognitive recovery (DNR)**: This term denotes cognitive decline diagnosed within 30 days of surgery. It supersedes the previous term, “early POCD”, and acknowledges the potential for cognitive function recovery.
**Postoperative NCD**: Cognitive decline identified between 30 days and 12 months after surgery.
The “postoperative” qualifier is no longer applied beyond the 12-month unless the diagnosis is established within this timeframe.

**Table 2:** Key terminology and definitions related to PNDs.

**Term**	**Definition**
Perioperative neurocognitive disorders (PNDs)	Cognitive deficits occurring before, during, and after surgery, encompassing delirium and cognitive dysfunction.
Preoperative NCD	Cognitive impairment before surgery is classified as mild or major based on severity and affects daily activities.
Postoperative delirium (POD)	Acute, fluctuating attention, awareness, and cognition disturbances typically occur within 1 week post-surgery.
Delayed neurocognitive recovery (DNR)	This term denotes cognitive decline diagnosed within 30 days of surgery.
Postoperative NCD	Cognitive decline was identified between 30 days and 12 months post-surgery.

The transition from POCD to PNDs aims to align perioperative cognitive impairment research with the clinical diagnostic criteria utilized in other medical fields, facilitating improved recognition and communication among experts across various disciplines. This updated nomenclature underscores the significance of a comprehensive assessment, including objective cognitive testing, evaluation of activities of daily living, and cognitive concerns reported by patients or informants.

In addition to the DSM-5 criteria, other neurocognitive assessment tools are used to evaluate the cognitive function of patients with PNDs. The Mini-Mental State Examination (MMSE) is a brief screening tool that assesses cognitive domains, such as memory, attention, language, and visuospatial skills. It is commonly used to detect cognitive impairment but may lack sensitivity for mild cognitive changes (13). The Montreal Cognitive Assessment (MoCA) is a more sensitive tool for detecting mild cognitive impairment. It assesses domains such as executive function, memory, and attention. It is particularly useful for identifying early cognitive decline in the surgical population (14). Trail Making Test (TMT) is a test that evaluates executive function, including cognitive flexibility and processing speed. It is often used to assess frontal lobe function (15). The Digit Span Test, part of the Wechsler Adult Intelligence Scale, assesses short-term memory and attention and can be used to diagnose PNDs (16). Furthermore, informant reports and functional assessments are crucial for diagnosing PNDs (17). Informant reports, such as those from family members or caregivers, provide valuable insights into patients' cognitive and behavioral changes. Functional assessments, such as the Activities of Daily Living (ADL) scale, evaluate a patient's ability to perform daily tasks and measure the impact of cognitive impairment on daily functioning (18).

The integration of these diagnostic tools and criteria allows for a more comprehensive evaluation of PNDs, facilitating early identification and intervention. Early diagnosis is critical for implementing targeted prevention and treatment strategies, improving postoperative outcomes, and enhancing patients' quality of life.

## 4 Risk factors and mechanisms

The reported frequency and occurrence of PNDs exhibit considerable variation, which is attributed to differences in the studied populations, surgical procedures, and diagnostic criteria used. Nevertheless, it is posited that a substantial proportion of patients, particularly older individuals, may experience some degree of cognitive deterioration following surgical interventions (19). A specific study found that cognitive impairment was observed in 53% of patients who underwent coronary artery bypass graft surgery, measured 5 years post-procedure. The study also noted that 36% of patients exhibited cognitive dysfunction at 6 weeks postoperatively, while 24% demonstrated impairment at 6-month mark (20).

Several factors influence the development and prevalence of PNDs. These contributing elements can be categorized into modifiable and non-modifiable factors, with both types playing a role in the onset of these disorders (Table 3). Understanding these factors is crucial for developing preventive strategies and personalized treatment approaches for patients undergoing surgery.

**Table 3:** Key Risk Factor Related to PNDs.

**Risk factor types**	**Risk factors**	**Description**
Non-modifiable	Age	Older adults are at higher risk for cognitive decline post-surgery.
	Genetic factors	Specific gene variations may increase susceptibility to PNDs.
	Preexisting medical conditions	Conditions like cardiovascular disease, respiratory disease, and diabetes raise the risk.
	Existing cognitive deficits	Pre-existing conditions like dementia or mild cognitive impairment make PND outcomes worse.
Modifiable	Surgical procedure complexity	More complex surgeries lead to higher risks of PNDs.
	Anesthetic techniques	The type and method of anesthesia may influence cognitive outcomes.
	Perioperative care practices	Maintenance of normal body temperature, blood pressure, and pain control can affect PND risk.
	Sleep management	Addressing sleep disturbances post-surgery is crucial to reduce the risk of PNDs.
	Others	Anti-inflammatory and aerobic exercise help improve PNDs.

### 4.1 Non-modifiable risk factors

Age is a crucial non-alterable risk factor for PNDs, with elderly individuals demonstrating heightened vulnerability to cognitive deterioration following surgical interventions (21). Aging can lead to a fragile brain, which can develop cerebrovascular lesions, altered cerebrospinal fluid flow, and waste accumulation (22), inducing neuroinflammation and causing altered cognitive function postoperatively (23).

Genetic predisposition may also affect the likelihood of experiencing PNDs. Studies have indicated that certain gene variants linked to inflammasome pathways are correlated with an elevated risk of developing inflammatory and neurodegenerative disorders, potentially facilitating the emergence of PNDs (24). In addition to these biological mechanisms, other genetic predispositions, specifically the presence of the apolipoprotein (APOE) ε4 allele, can further modulate an individual's susceptibility to PNDs (25). Advances in genomic research may uncover distinct genetic markers or variations that increase the risk of PNDs, paving the way for customized preventive strategies and therapeutic interventions (24, 25).

Preexisting medical conditions, such as cardiovascular disease, respiratory disease, and diabetes, can increase the risk of PNDs due to physiological stress during the perioperative period (26). Magnetic resonance imaging (MRI) studies have linked decreased hippocampal volume to PNDs, indicating that cerebrovascular factors may play a role in their development (27). Cerebrovascular changes significantly affect the pathophysiology of PNDs and contribute to cognitive impairment. A large-scale retrospective cohort study of 13,899 surgical patients investigated the relationship between pre-existing cerebrovascular disease (CVD) and PNDs. The findings revealed that CVD is an independent risk factor for PNDs, suggesting a strong association (28).

Furthermore, existing preoperative cognitive deficits, such as dementia or mild or major cognitive impairment, can increase the likelihood of PNDs and adversely affect their outcomes. Research indicates that cognitive exercises performed before surgery may enhance cognitive function post-procedure, potentially reducing the incidence of PNDs (9).

### 4.2 Modifiable risk factors

PNDs are significantly influenced by the complexity and invasiveness of surgical interventions, with more intricate procedures generally associated with a higher likelihood of occurrence. Surgical operations can induce fluctuations in blood pressure and cerebral blood flow (CBF), potentially leading to ischemia-reperfusion injury and compromising the integrity of the blood-brain barrier (BBB) (29). Such physiological changes can result in neuronal damage, particularly in vulnerable regions, such as the hippocampus, which is crucial for memory function. The nature of the surgical procedure is also critical, as more invasive operations, especially those involving the heart or major abdominal areas, are often correlated with an increased risk of PNDs (30). Evidence suggests that surgical trauma in peripheral tissues can trigger an inflammatory response, releasing damage-associated molecular patterns (31) and inflammatory cytokines such as IL-1β, IL-6, and TNF-α. These cytokines may compromise the BBB, allowing inflammatory mediators to infiltrate the central nervous system (CNS). This infiltration can initiate neuroinflammation, leading to neuronal dysfunction and subsequent cognitive decline (11). Most experts consider neuroinflammation to be a key factor in the pathomechanism of PND (32).

Perioperative care practices during surgical interventions, including anesthetic agent selection, anesthetic depth, normothermia maintenance, and blood pressure monitoring, can significantly impact the incidence of PNDs (33). The choice of anesthetic agent influences postoperative cognitive outcomes. Inhaled anesthetics, such as isoflurane and sevoflurane, have been linked to an increased risk of postoperative cognitive dysfunction (POCD) in elderly populations (34). These agents may induce neuroinflammation and compromise BBB integrity, leading to cognitive decline in older adults. Conversely, intravenous anesthetics such as propofol and dexmedetomidine have potential neuroprotective effects. Propofol can reduce neuroinflammation and oxidative stress, potentially mitigating cognitive deterioration (35). Dexmedetomidine, an α2-adrenergic agonist, has been found to enhance glymphatic function and decrease the incidence of delirium and cognitive impairment in older patients (22, 36). While anesthetic neurotoxicity remains debated, some studies suggest that these substances may directly affect neuronal function and survival (37). Additionally, anesthetics can interact with neurotransmitter systems, particularly the cholinergic system, which is crucial for cognitive functions such as memory and attention (38). Anesthesia-induced disruptions in neurotransmitter systems may contribute to post-surgery cognitive deficits (39).

Depth of anesthesia is another critical factor influencing cognitive outcomes. Studies have demonstrated that excessively deep anesthesia, as measured by the Bispectral Index (BIS), is associated with an increased risk of PNDs. Maintaining an appropriate depth of anesthesia, guided by BIS monitoring, can help reduce the risk of cognitive decline during surgery. Additionally, multimodal anesthesia, which combines different anesthetic agents and techniques, has improved cognitive outcomes by minimizing the dose of individual agents and reducing their adverse effects (33).

Perioperative hemodynamic management is crucial for reducing the risk of PNDs. Hypotension and blood pressure fluctuations during surgery can lead to cerebral hypoperfusion, increasing the risk of cognitive decline. Maintaining stable hemodynamics through careful monitoring and the use of vasoactive agents, such as norepinephrine and phenylephrine, can help preserve CBF and reduce the risk of PNDs (29).

Postoperative pain management is a modifiable risk factor. Poor pain control can cause sleep disturbances, stress, and neuroinflammation, all of which contribute to the cognitive decline. Effective pain management using opioids and non-opioid analgesics can reduce the risk of PND (40). Post-surgery sleep disruptions increase neuronal activity and produce waste products, such as lactate, which are cleared through glymphatic fluid transport (41, 42). These disturbances can impair the waste-clearing efficiency of the glymphatic system, leading to amyloid-beta (Aβ) accumulation in critical brain regions, including the thalamus and medial temporal areas (22, 42). Sleep meliorating through pharmacological and non-pharmacological interventions, such as melatonin or cognitive-behavioral therapy, can improve sleep quality and lower the risk of cognitive decline (40).

PNDs arise from multiple factors, including neuroinflammation, cerebrovascular changes, and anesthetic effects, which are not yet fully understood. Ongoing research is vital for developing strategies to reduce the occurrence and severity of PND in surgical patients, as it is influenced by patient and surgical factors. Understanding these interactions is key to improving patient care and outcomes in the perioperative phase.

## 5 Prevention and management strategies

Preventing and managing PNDs is essential for minimizing surgical risks, especially in older patients. Both drug- and non-drug-based approaches have been investigated to address postsurgical cognitive decline. Non-pharmacological methods have gained attention because of their potential to enhance cognitive function and overall wellbeing without the side effects associated with medication.

Physical activity has emerged as a key non-drug strategy for reducing the occurrence and intensity of PNDs (43). A recent literature review emphasized that exercise may improve cognitive dysfunction-related conditions, including PNDs, through various mechanisms, such as reducing neuroinflammation, influencing gut bacteria, preserving muscle mass, improving mitochondrial function, and affecting synaptic plasticity (44, 45). Animal studies on swimming and running have demonstrated a reduction in inflammatory proteins and changes in the gut microbiota, leading to improved cognitive function after surgery (44).

Preventive strategies include cognitive training, optimized anesthesia, and cognitive rehabilitation. Effectiveness is evaluated through RCTs, longitudinal studies, and meta-analyses, showing that cognitive training improves postoperative function in elderly patients (20, 46, 47). However, variability in study designs and outcomes presents challenges, underscoring the need for standardized protocols to facilitate comparisons.

Pharmacological approaches aim to target the specific biological mechanisms involved in PNDs. However, it is worth noting that drug-based management of PNDs is complex because of the diverse nature of these disorders. The medications studied include those targeting inflammation (36–38), dexmedetomidine (36), neurotransmitter activity (48), and neuroprotection (49). For instance, drugs that inhibit acetylcholinesterase and block NMDA receptors have been explored for PNDs, although evidence remains limited, and treatment efficacy has not been consistently demonstrated (50).

Owing to the diverse nature of PNDs, personalized treatment strategies are essential. These strategies should consider the patient's pre-surgery cognitive condition, health issues, surgical procedure, anesthesia management, and individual preferences (51). Ongoing cognitive evaluations can assist in tracking improvements and modifying the treatment approach as required. A comprehensive rehabilitation plan incorporating physical, occupational, and cognitive-behavioral therapies may be advantageous in addressing PNDs (52). This holistic approach can target various aspects of recovery, ranging from physical capabilities and movement to mental and emotional health. Addressing PNDs effectively requires a multi-pronged strategy encompassing non-drug interventions, such as physical activity, medications targeting the underlying mechanisms, and tailored care plans that adapt to the patient's evolving needs and progress. Additional studies are required to determine the most successful approach to enhance patient outcomes.

## 6 Future research directions

PNDs present a complex challenge with notable knowledge gaps in neurology, anesthesiology, geriatrics, and psychiatry. Although researchers have explored aspects such as neuroinflammation and the effects of anesthetics, the underlying processes remain poorly understood. Interdisciplinary collaboration has the potential to advance diagnostic and preventive treatment strategies.

Advanced noninvasive brain imaging techniques have emerged as powerful tools for understanding the structural and functional changes associated with PNDs. Functional magnetic resonance imaging (fMRI) and positron emission tomography (PET) provide insights into brain alterations linked to PNDs (53, 54). fMRI evaluates cerebral activity by monitoring blood oxygenation and the perivascular space, highlighting the surgical and anesthetic effects on neural circuits (55). It reveals functional brain changes, especially in hippocampal and prefrontal cortex connectivity. Fislage et al. also showed disrupted connectivity in patients with postoperative delirium (56). Diffusion tensor imaging (DTI) assesses white matter integrity, aiding in the detection of connectivity disruptions that lead to cognitive decline (57). PET reveals metabolic changes in regions associated with memory and cognition, such as the hippocampus and prefrontal cortex, while amyloid accumulation detected by PET is linked to the intensity of perioperative delirium (58). Other noninvasive brain stimulation methods, including transcranial magnetic stimulation (TMS) and transcranial direct current stimulation (tDCS), show promise for PNDs treatment. TMS uses magnetic fields to activate brain regions, potentially enhancing cognition (59), while tDCS applies a low electrical current to stimulate areas, possibly improving cognitive performance and alleviating PND symptoms (60). These approaches offer potential diagnostic and preventive treatment strategies.

The integration of machine learning and artificial intelligence (AI) in neuroimaging data analysis is an exciting area of research. Machine learning algorithms analyze complex fMRI, PET, and DTI datasets to identify patterns and biomarkers associated with PNDs (61). Predicting postoperative delirium by analyzing preoperative risk factors and intraoperative information highlights the potential of machine learning for tailored risk evaluations and targeted interventions. AI-driven models can predict cognitive outcomes based on preoperative and postoperative imaging data, enabling the early identification of at-risk patients and personalized interventions (62). The application of machine learning and AI to complex datasets from neuroimaging, genetic research, and electronic health records may uncover new patterns and predictors of PNDs and personalized treatment responses. These technologies could revolutionize PND diagnosis and management by providing more accurate and timely assessments of cognitive function.

Future studies should explore the intricate molecular and cellular mechanisms underlying PNDs.

The discovery of biological indicators in the blood or cerebrospinal fluid that indicate the presence or likelihood of PNDs could facilitate early detection and treatment monitoring. These indicators may include proteins and other biochemical markers associated with neuroinflammation and neuronal injury (63). Advanced techniques may facilitate the identification of biological markers for the early detection and monitoring of cognitive decline, particularly in patients with MCI who are often overlooked.

The development of advanced cognitive assessment tools, including virtual reality and mobile applications, may provide more sensitive measures of cognitive function, allowing for better monitoring of PNDs and intervention effects (64). Investigating the impact of lifestyle elements, such as nutrition, rest, and physical activity, on PNDs risk could provide recommendations for preoperative preparation to reduce cognitive decline (65). As our knowledge deepens, innovative technologies and methodologies are expected to emerge, fostering optimism about improved patient outcomes.

## 7 Clinical implications

PNDs pose significant challenges in clinical practice, impacting patient recovery with extended hospital stays, increased health care costs, and reduced quality of life. Clinical manifestations include cognitive, behavioral, and emotional changes, such as memory impairment, disorientation, slowed thinking, attention deficits, agitation, irritability, and mood swings.

Diagnosing PNDs requires a multifaceted approach involving clinical assessment, cognitive testing, and exclusion of other potential causes of cognitive impairment. The DSM-5 provides a framework for diagnosing neurocognitive disorders; however, its criteria may not fully capture perioperative cognitive changes.

Enhanced perioperative and postoperative care is crucial for mitigating the incidence of PNDs. Preoperatively, cognitive assessment tools such as the MMSE and MoCA should be used to identify at-risk patients. Optimizing comorbid conditions, such as hypertension and diabetes, is vital for reducing cognitive risks.

In the postoperative phase, care should prioritize early identification and intervention. Regular cognitive assessments are recommended using tools such as the Confusion Assessment Method for delirium and MoCA for cognitive dysfunction. The implementation of cognitive rehabilitation programs, including memory training and attention exercises, can enhance cognitive function (66). In selected cases, pharmacological interventions such as cholinesterase inhibitors, NMDA receptor antagonists, and dexmedetomidine may be considered, although evidence supporting these treatments is limited. The integration of these approaches can improve patient outcomes and reduce the incidence of PND (50).

A multidisciplinary strategy is crucial for managing PNDs and requires expert collaboration to develop personalized care plans. Educating patients about PND risks and cognitive health strategies is essential for proactive care and improving patient outcomes. A lower socioeconomic status limits healthcare access, worsens comorbidities, and deteriorates postoperative cognitive outcomes (67). Cultural factors influence care-seeking behaviors, treatment adherence, and symptom reporting (68). Understanding perioperative cognitive changes while committing to education, collaboration, and research, improves patient care.

## 8 Controversy over the term of PNDs

The concept of “PNDs” encompasses various cognitive issues and has sparked significant debate and disagreement within the medical field, largely due to the intricacies involved in characterizing, identifying, and comprehending the underlying processes of these cognitive alterations experienced by surgical patients (69). Skeptics contend that this broad classification may result in ambiguity and potentially obscure the fundamental mechanisms and distinct clinical manifestations of these conditions. A unified diagnostic approach is essential for enhancing the credibility of research outcomes and clinical methodologies (1). There is a demand for more precise differentiation between various cognitive impairments and their specific attributes. Some individuals may not display indications of cognitive dysfunction until after their surgical procedure, making it challenging to detect and address these issues. The diversity of research methodologies, subject demographics, and outcome assessments complicates the integration of findings and restricts the ability to draw robust conclusions that could influence medical practice. Although factors such as inflammation, anesthetic-induced neurotoxicity, and vascular changes have been suggested, the exact mechanisms remain unknown.

Assigning PNDs diagnoses to patients can result in stigma toward both patients and their families. The fear of being categorized as having cognitive impairment post-surgery may prevent individuals from seeking the necessary assistance or support, potentially impacting their recovery and overall wellbeing (70). The term “PNDs” might confuse patients and the public, potentially causing anxiety about surgery-related cognitive decline. To address fears and misunderstandings, improving communication and education about PNDs, their associated risks, and the nature of post-surgical cognitive changes is essential. The debates surrounding “PNDs” underscore the need for continued research, better communication, and cooperation among medical professionals to address the intricacies of postsurgical cognitive alterations. As this field progresses, more precise terminology and diagnostic standards may be developed, which will help inform medical practice and patient care.

## 9 Conclusion

This review describes the changing nomenclature and multiple risk factors for PNDs and considers variables such as patient age, type of surgery, pre-existing medical conditions, and perioperative nursing practices. This emphasizes the need for comprehensive strategies to manage PNDs, including enhanced perioperative protocols, patient awareness initiatives, and shared decision-making, to reduce the risk and promote recovery. Effectively addressing PNDs in clinical settings requires a preemptive stance on prevention and treatment by medical professionals who implement research-supported strategies. This encompasses thorough preoperative evaluations, appropriate anesthetic technique selection, and the adoption of postoperative care guidelines prioritizing cognitive wellbeing. Furthermore, this study advocates for ongoing scientific exploration and advancement to uncover efficacious interventions and enhance our understanding of PNDs. By broadening our knowledge base and refining clinical practices, the healthcare community can effectively tackle the challenges associated with PNDs and enhance perioperative outcomes. Collaborative efforts among healthcare providers, researchers, and patients are crucial for fostering a clinical environment that reduces the occurrence and impact of PNDs.
